# Analysis of Collapse Strength and Life Prediction of Casings in Formation Water Service Environments

**DOI:** 10.3390/ma18132934

**Published:** 2025-06-20

**Authors:** Wanzhong Li, Jinlong Fan, Pengbo Huo, Yongqiang Zhang

**Affiliations:** 1 School of Mechanical Engineering, Xi’an Shiyou University, Xi’an 710065, China; liwanzhong@xsyu.edu.cn (W.L.); pbhuoxsy@163.com (P.H.); 2 Research Institute of Shaanxi Yanchang Petroleum (Group) Co., Ltd., Xi’an 710075, China; zhangyqslb@163.com

**Keywords:** casing corrosion, service life prediction, pitting corrosion, volume loss, finite element analysis

## Abstract

In oil and gas field development, the highly mineralized formation water environment often leads to casing corrosion, significantly reducing its collapse strength and service life. To investigate the corrosion characteristics and mechanical degradation of casings in formation water, this study combines high-temperature high-pressure (HTHP) corrosion experiments with finite element analysis to examine the influence of corrosion pit geometry and volume loss on casing collapse strength. Based on experimental data and simulation results, the safe service life of casing under various conditions is also predicted. The results show that greater material loss due to corrosion leads to lower collapse strength. Under the same volume loss, cylindrical pits cause the most severe reduction in collapse strength, while semi-ellipsoidal pits result in the least degradation. Elevated temperature and pressure significantly accelerate corrosion and further reduce mechanical performance. Under service conditions of 20 MPa–80 °C, 11 MPa–50 °C, and 2 MPa–20 °C, the predicted safe service life of the casing is 4.84, 7.57, and 11.61 years, respectively. The approach proposed in this study provides a reference for the evaluation of collapse resistance and service life prediction of casings exposed to formation water environments.

## 1. Introduction

In oil and gas field development, casings serve as key structural components supporting the wellbore wall, and their integrity directly affects wellbore stability and operational safety. However, casings are exposed to complex environments for extended periods, where high temperature, pressure, chemical media, and mechanical loads can collectively induce corrosion failures. Formation water, rich in Cl^−^, SO₄^2−^, HCO₃^−^, and microorganisms, significantly accelerates the corrosion process, leading to material degradation and reduced collapse strength of casing steel [1,2,3]. With increasing well depth, formation pressure also rises. When the casing strength becomes insufficient to resist the external load, collapse deformation or even structural failure may occur, posing a threat to well integrity and operational safety [4,5,6,7]. Therefore, accurately predicting casing collapse strength and service life is crucial for ensuring the safe and continuous production of oil and gas wells.

Dou et al. [8] conducted high-temperature and high-pressure (HTHP) flow experiments to simulate the corrosion behavior of N80 steel in downhole environments. Their findings showed that corrosion in oxygen- and CO₂-containing media was significantly influenced by temperature and gas concentration. Zhou et al. [9] experimentally studied the effects of CO₂/H₂S partial pressure, applied stress, and corrosion duration on corrosion rate, and proposed a multi-factor corrosion rate model. Li et al. [10] investigated the corrosion behavior of P110 casing steel in simulated concrete fluid and CO₂-saturated annular fluid, observing significant localized corrosion in both environments. Dong et al. [11] investigated the corrosion rates of N80 and P110 steels exposed to CO₂ environments and found that the corrosion rate of P110 was slightly higher than that of N80. Cheng et al. [12] studied the corrosion behavior of J55 and N80 steels in simulated formation water under different CO₂ partial pressures, and their results indicated that J55 exhibited generally lower corrosion rates than N80, suggesting better corrosion resistance.

Localized corrosion is one of the main causes of casing strength degradation. The shape, size, and distribution of corrosion pits have a critical influence on the residual collapse strength. Yan et al. [13] used ANSYS to build casing models with double-ellipsoidal pits and quantitatively analyzed the effects of pit depth, cross-sectional parameters, and pit spacing on collapse strength. Li et al. [14] modeled pipes with irregular pits and demonstrated that simplified volume-loss models are valid when actual and ideal volume losses are comparable. Sedmak et al. [15] evaluated the collapse resistance of damaged casings through experimental and numerical approaches, showing that the morphology and depth of pitting corrosion directly affect collapse performance. Wang et al. [16] developed a 3D nonlinear finite element model to study collapse pressure in randomly corroded pipelines. Nakai et al. [17] observed cylindrical, conical, and semi-ellipsoidal pit shapes in marine structures. Sheng et al. [18] simulated pitting corrosion through mechanical drilling and analyzed its impact on the tensile properties of steel, finding that fracture tends to occur at cross-sections with higher pit severity. Wang et al. [19] proposed a numerical method for evaluating residual strength based on corrosion morphology, using finite element modeling of corroded surfaces. Wang et al. [20] used Python and ABAQUS to assess the influence of randomly distributed local pits on the collapse pressure of 2D rings, revealing that pit depth is the dominant factor. Dobson et al. [21] scanned three types of corrosion pits and converted them into FE models to analyze the stress concentration factor (*k_t_*), finding that real pit geometries can produce *k_t_* values 1.32–1.65 times higher than ideal semi-ellipsoidal pits.

Common methods for pipeline life prediction include machine learning approaches, probabilistic statistical methods, and empirical formula-based models. Li et al. [22] applied six machine learning algorithms to predict casing failure probability under the coupling effects of multiple factors. The results showed that the Random Forest and LightGBM models exhibited the best generalization performance. Hu et al. [23] used the Monte Carlo method to simulate the stochastic and statistical characteristics of crack growth over time, thereby estimating the residual life of pipelines. Ossai et al. [24] employed a continuous-time non-homogeneous linear growth pure-birth Markov model combined with field data to predict the distribution of corrosion pit depths in oil and gas pipelines. El Amine Ben Seghier et al. [25] developed a hybrid intelligent model by integrating empirical formulas with machine learning to predict the maximum pit depth in oil and gas pipelines.

Few studies have integrated actual formation water conditions to predict casing service life. In this study, J55-grade casing was selected, and its corrosion behavior under simulated formation water conditions was investigated using an HTHP autoclave. Finite element models with different pit geometries were developed to evaluate the effect of pit shape and volume loss on collapse strength. Combined with experimental and simulation results, casing service life under varying temperature and pressure conditions was predicted. This study provides a reference for evaluating collapse resistance and service life of casing in formation water environments.

## 2. Materials and Methods

In this study, the corrosion behavior of casing in formation water environments was simulated using a high-temperature and high-pressure (HTHP) autoclave, aiming to evaluate the effects of corrosion on the mechanical properties of casing. The experimental program consisted of two parts: corrosion testing using hanging coupon specimens and full-scale casing specimens.

### 2.1. Materials and Specimen Preparation

The material used in the experiment was J55-grade casing steel, with an outer diameter of 139.7 mm and a wall thickness of 7.72 mm. Its chemical composition is listed in Table 1. Tensile tests were conducted using a universal testing machine at the Shaanxi Black Metallurgical Products Quality Supervision and Inspection Station (Xi’an, China) to determine the yield strength, ultimate tensile strength, and elongation of the casing material. In addition, Rockwell hardness tests were performed at the same institution to assess the material hardness. The measured mechanical properties are summarized in Table 2.

**Figure 1:**
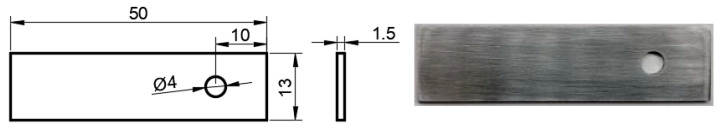
Appearance and dimensions of the hanging coupon specimen.

In accordance with the SY/T 6128-2012 standard [26], both full-scale casing specimens and hanging coupon specimens were prepared. Three full-scale casing specimens, each with a length of 1500 mm, were fabricated for evaluating collapse resistance under corrosive conditions. Hanging coupon specimens were used to analyze corrosion morphology, pitting characteristics, and corrosion rate. Three groups of hanging coupons were prepared, with their dimensions and geometry shown in Figure 1. All specimens were subjected to degreasing, cleaning, and drying prior to testing to eliminate the influence of surface contaminants on corrosion behavior.

### 2.2. Corrosion Test of Hanging Coupon Specimens

To simulate the service conditions of casing under different well depths, three typical temperature–pressure conditions were designed based on field data from the Yanchang Oilfield, Xi’an, Shaanxi, China, 20 MPa–80 °C, 11 MPa–50 °C, and 2 MPa–20 °C, corresponding to the actual service environments of the deep well section, the middle well section, and the wellhead section, respectively.

The corrosion medium was prepared according to the ionic concentrations measured from field formation water samples. Specifically, 126.97 mg of NaHCO_3_, 1308.91 mg of Na_2_SO_4_, 506.55 mg of MgCl_2_, 37560.81 mg of NaCl, and 3512.84 mg of CaCl_2_ were added to every 1000 mL of purified water to formulate the simulated formation water solution.

The hanging coupon test was conducted to quantitatively analyze the corrosion rate and pitting morphology of the casing material. According to the GB/T 17897-2016 standard [27], prior to testing, the six working surfaces of each specimen were sequentially polished using 200#, 400#, 800#, and 1200# metallographic abrasive papers until a smooth and uniform surface was obtained. All specimens were then thoroughly cleaned, dried, weighed using an electronic balance, and photographed for documentation. During the experiment, the specimens were placed in a high-temperature and high-pressure autoclave. Based on the autoclave volume, the corrosion solution was added proportionally to ensure that each square centimeter of exposed specimen surface had no less than 20 mL of corrosion solution. Heating and pressurization were conducted under the conditions specified in Table 3, and the exposure duration was set to 7 days.

After the experiment, the corroded specimens were photographed to record their surface condition and corrosion product adhesion. The samples were then immersed in a cleaning solution for 10 min to remove corrosion products, and gently wiped clean with degreased cotton. After drying, the specimens were reweighed using the electronic balance, and the mass loss was recorded. According to the approach presented in [11], the corrosion rate was calculated using Equation (1). Subsequently, a metallographic microscope was employed to examine the microscopic surface morphology, including the shape, number, and distribution of pits. A pitting depth gauge was used to measure the maximum depth of corrosion pits, and the number of pits per unit area was statistically analyzed.

(1) $$C_{r}=\frac{8.76\times 10^{7}\times \left(M-M_{1}\right)}{S\times T\times D}$$

where *C_r_* is the corrosion rate, in millimeters per year (mm/a), *M* and *M*₁ are the initial and final mass of the specimen (g), *S* is the surface area (cm^2^), *T* is the exposure time (h), and *D* is the material density (8000 kg/m^3^).

### 2.3. Full-Scale Casing Corrosion Test and Collapse Strength Evaluation

**Figure 2:**
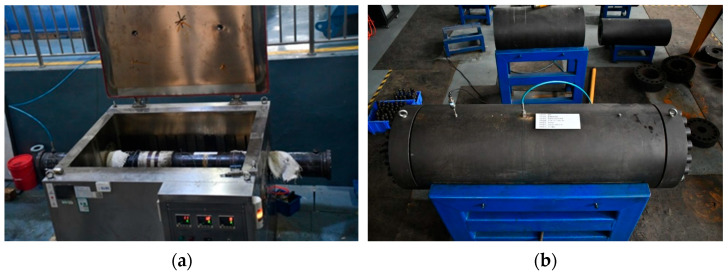
Experimental apparatus: (**a**) high-temperature and high-pressure autoclave; (**b**) casing collapse and burst testing machine.

The full-scale casing specimens were placed into a high-temperature and high-pressure autoclave, as shown in Figure 2a. A pre-prepared simulated formation water solution was used as the corrosion medium. Three corrosion conditions, with varying temperatures and pressures, were designed to investigate the influence of environmental factors on the corrosion behavior and mechanical performance of the casing. The exposure period was 30 days, and the detailed parameters are listed in Table 3.

After the corrosion test, the casing specimens were removed from the autoclave, cleaned, and dried. The corrosion solution in the annular space between the casing and the autoclave was also discharged. Subsequently, external pressure collapse tests were conducted on the corroded full-scale casing specimens using the casing collapse and burst testing apparatus (Xi’an Maurer Petroleum Engineering Laboratory Co., Ltd., Xi’an, China), as shown in Figure 2b. During the test, the corroded casing specimen was placed inside the pressure chamber, and a sealed pressure cavity was formed by tightening the sealing components at both ends to allow the application of the pressurizing medium. The testing apparatus offers high measurement accuracy, with a pressure control error of less than 1% and a pressurization rate of 0.17 ± 0.02 MPa/s. Water was used as the pressurizing medium, and pressure was applied at a constant rate. The ultimate external pressure at which the casing collapsed was recorded.

### 2.4. Finite Element Model of Casing with Corrosion Defects

To investigate the influence of pit geometry and volume loss on the collapse strength of casing, a finite element model (FEM) incorporating corrosion pits was developed using ABAQUS. Since actual corrosion pit shapes on the casing surface are complex and difficult to model accurately, the following assumptions were made to improve computational efficiency:

(1) The shape of the corrosion pits on the casing surface is simplified to regular cylindrical, conical, and semi-ellipsoidal forms, and these pits are uniformly distributed along the central area of the casing’s outer wall.
(2) The material is assumed to be homogeneous and isotropic.

The model dimensions and mechanical performance parameters are based on full-scale casing sample specifications. To avoid high stress concentration at the ends of the casing, MPCs (multi-point constraints) are applied at both ends of the casing. A uniform increased external pressure is applied to the middle section of the outer wall. The Riks method is used to trace the instability path, and the critical pressure at instability is extracted as the casing’s anti-collapse strength [28,29]. The mesh is primarily generated using hexahedral elements, with mesh refinement at the corrosion pit locations. At least four layers of elements were used along the casing wall thickness direction. If fewer layers of mesh are used in the wall thickness direction, it may lead to significant errors in the calculation results.

## 3. Results and Discussion

### 3.1. Influence of Temperature and Pressure on Casing Corrosion Behavior

**Figure 3:**
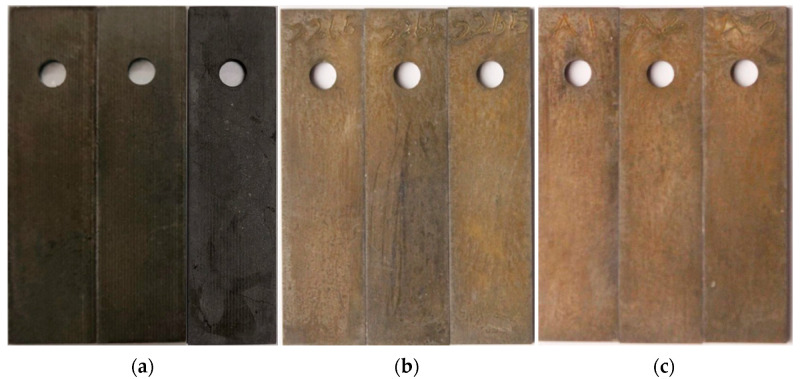
Macroscopic surface morphology of casing after corrosion: (**a**) 2 MPa–20 °C; (**b**) 11 MPa–50 °C; (**c**) 20 MPa–80 °C.

The macroscopic surface morphology of the corroded specimens is shown in Figure 3. Significant differences in surface corrosion product attachment can be observed under different conditions. Under the conditions of 20 MPa–80 °C and 11 MPa–50 °C, a large amount of yellow-brown corrosion product formed on the specimen surfaces. In contrast, only a small amount of corrosion product was observed under the 2 MPa–20 °C condition. These results indicate that increasing temperature and pressure accelerates the corrosion rate of the casing material.

**Figure 4:**
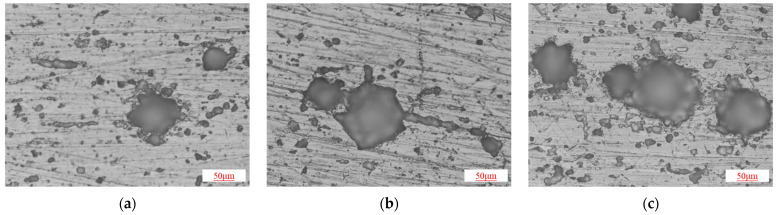
Microscopic surface morphology of casing after corrosion: (**a**) 2 MPa–20 °C; (**b**) 11 MPa–50 °C; (**c**) 20 MPa–80 °C.

Subsequently, the microstructural corrosion morphology of the specimen surface was examined using a metallographic microscope. If pitting corrosion was observed, the pit depth was measured using a pit depth gauge. Figure 4 presents the surface corrosion morphology of the specimens at a 50 μm scale. Various pitting shapes were observed on the specimen surfaces, and the two-dimensional projections of the pits were generally approximately circular. Under the 20 MPa–80 °C condition, the specimen surface exhibited severe localized corrosion, whereas under the 2 MPa–20 °C condition, only mild localized corrosion was observed.

Table 4 presents the measured and calculated average corrosion rates, maximum pit depths, and pit densities. The results demonstrate that both maximum pit depth and pit density increase with rising temperature and pressure. Compared to 2 MPa–20 °C, the average corrosion rate under 11 MPa–50 °C increased by 87.5%, while under 20 MPa–80 °C it only increased by an additional 13.3%. This indicates that although corrosion rate generally increases with temperature and pressure, the rate of increase tends to slow once a certain threshold is surpassed.

### 3.2. Collapse Strength of Corroded Casing and Model Validation

**Figure 5:**
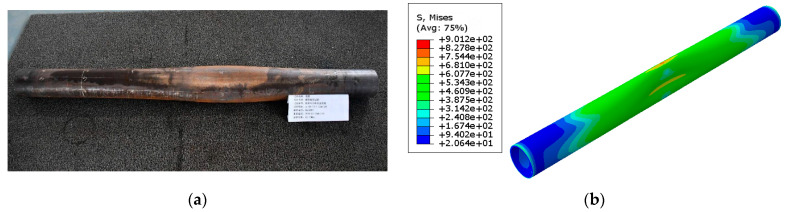
Casing collapse test results under the 11 MPa–50 °C condition. (**a**) Photograph of the casing after collapse; (**b**) stress contour of the casing at the point of collapse.

The collapse strength of the corroded full-scale casing was tested using a casing collapse and burst testing machine. The test results are presented in Table 5, and the post-collapse condition of the specimen is shown in Figure 5a.

The findings show that casing collapse strength decreased with increasing temperature and pressure. At 20 MPa–80 °C, the lowest collapse strength was recorded at 43.37 MPa, a decrease of approximately 1.3% compared to the 2 MPa–20 °C condition. This trend is consistent with that of corrosion rate: higher temperatures and pressures accelerate corrosion, reduce wall thickness, and consequently diminish collapse strength.

Finite element simulations of casing with corrosion defects revealed instability phenomena once the external pressure exceeded a certain threshold, as shown in Figure 5b. The Riks method was used to trace the post-buckling path and determine the critical collapse pressure. The simulation results were compared with experimental values, showing a maximum error of less than 4%. The model accurately captured the degradation of collapse strength with increasing corrosion severity, confirming that the FEM effectively simulates the mechanical weakening effect of corrosion. The minor discrepancies can be attributed to the following:

(1) The corrosion defects modeled in the finite element simulation differ from the actual defect morphology;
(2) The casing was modeled as having a perfectly circular cross-section, and the influence of ovality on collapse strength was not considered;
(3) The simulation of the plastic deformation stage involves complex nonlinear behavior, leading to deviations from the actual test results.

### 3.3. Influence of Pit Geometry on Collapse Strength

The hanging coupon corrosion tests revealed that most corrosion pits exhibit approximately circular two-dimensional projections. For modeling and analysis purposes, the pit shapes in this study were simplified as cylindrical, conical, and semi-ellipsoidal. All other parameters were kept constant across the models except for pit shape.

**Figure 6:**
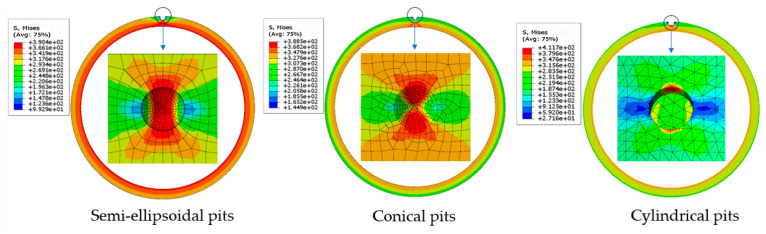
Equivalent stress distribution of different pit shapes at casing yield.

Figure 6 illustrates the stress distribution for different pit geometries. Corroded areas severely weakened the load-bearing capacity of the casing, and stress concentration at pit sites led to early yielding. Long-term loading may propagate these local weaknesses, compromising the overall strength. Semi-ellipsoidal pits have smooth U-shaped cross-sections, resulting in a more uniform stress distribution. Cylindrical pits have a sharp L-shaped bottom that causes high stress concentrations along the vertical edges and inner walls. Conical pits have V-shaped cross-sections, leading to uniform internal stress distribution but significant concentration at the tip.

**Figure 7:**
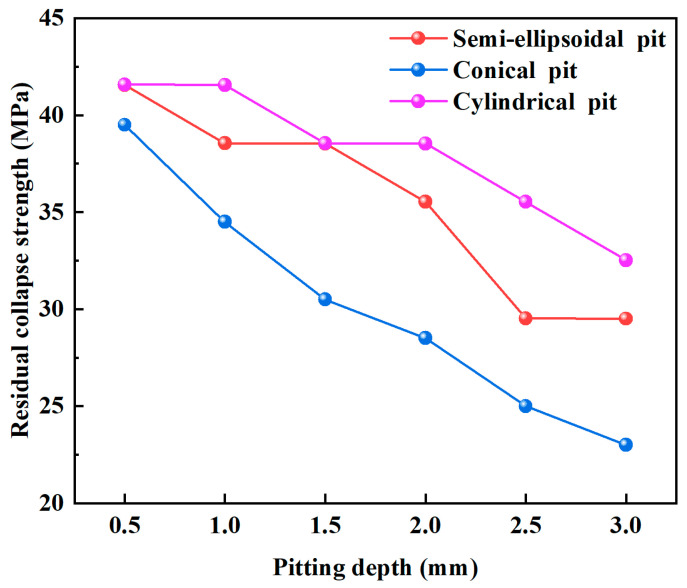
Influence of corrosion pit shape on the collapse strength of casing.

Figure 7 illustrates the effects of three corrosion pit shapes on the collapse strength of the casing at a constant pit diameter of 4 mm under varying pit depths. With increasing pit depth, residual collapse strength declined significantly. Cylindrical pits caused the greatest reduction, followed by semi-ellipsoidal and then conical pits. When the pit depth exceeded 2 mm, both semi-ellipsoidal and conical pits led to a more rapid decline in strength. This is due to geometric transitions—shallow ellipsoidal pits become deeper and more hemispherical, while conical pits become sharper with more acute tip angles, enhancing stress concentration.

Even when the pit diameter and depth are the same, different pit geometries cause distinct reductions in collapse strength, likely due to varying degrees of volume loss. Cylindrical pits resulted in the largest volume loss and thus the greatest strength degradation, while conical pits caused the smallest volume loss and had the least effect.

### 3.4. Influence of Volume Loss on Collapse Strength

**Figure 8:**
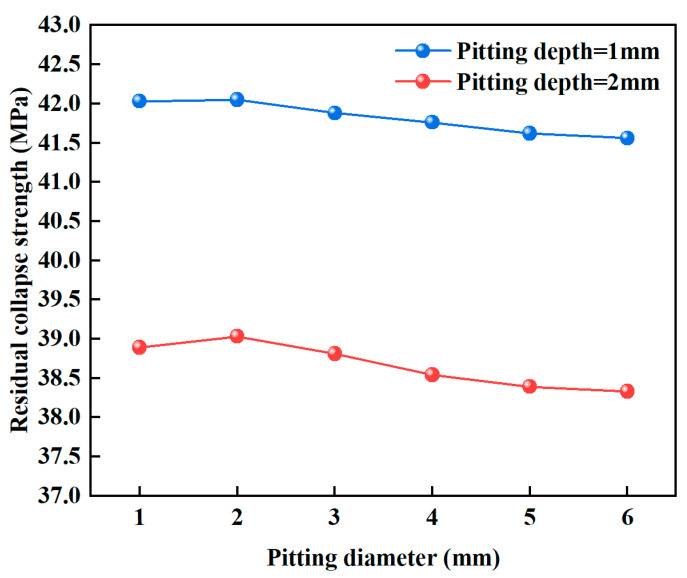
Influence of corrosion pit diameter on the collapse strength of casing.

The volume loss caused by corrosion pits leads to varying degrees of wall thickness reduction in the casing, which in turn affects its collapse resistance. In the analysis, the depth of all corrosion pits was kept constant, while the pit diameter was adjusted to ensure that the three different pit shapes had the same volume. To eliminate the influence of pit diameter differences on the results, conical pits were used as an example to investigate the trend of casing collapse strength with varying pit diameters. As shown in Figure 8, the collapse strength of the casing generally decreased with increasing pit diameter, but the magnitude of the change was relatively small. When the pit depths were 1 mm and 2 mm, increasing the diameter from 1 mm to 6 mm resulted in a reduction in collapse strength of only 0.47 MPa and 0.56 MPa, respectively. Therefore, compared to pit depth, the effect of pit diameter on the collapse resistance of the casing is relatively limited and can even be considered negligible.

**Figure 9:**
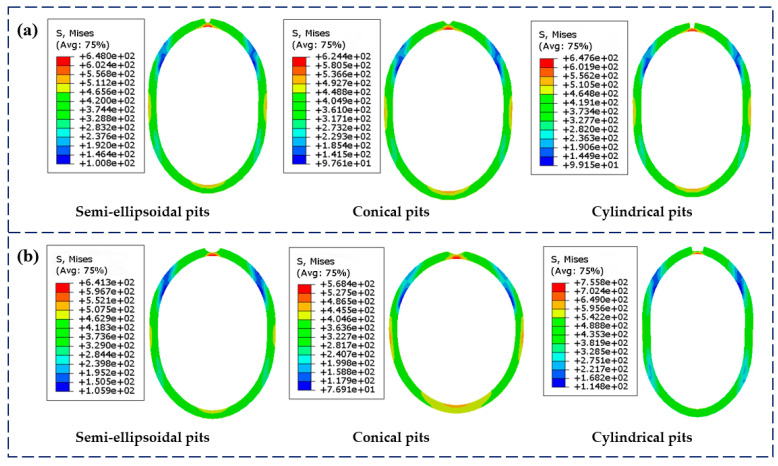
Equivalent stress contour of casing with different pit shapes at 100 mm^3^ and 500 mm^3^ volume loss: (**a**) 100 mm^3^ volume loss; (**b**) 500 mm^3^ volume loss.

Figure 9 shows the equivalent stress contours of the casing, indicating that collapse consistently occurs at the corrosion pit locations. This is attributed to high stress concentration and wall thinning caused by the pits, which lead to premature structural instability. It can also be observed that larger pit volumes result in greater volume loss, more severe wall thickness reduction, and consequently lower collapse strength of the casing.

**Figure 10:**
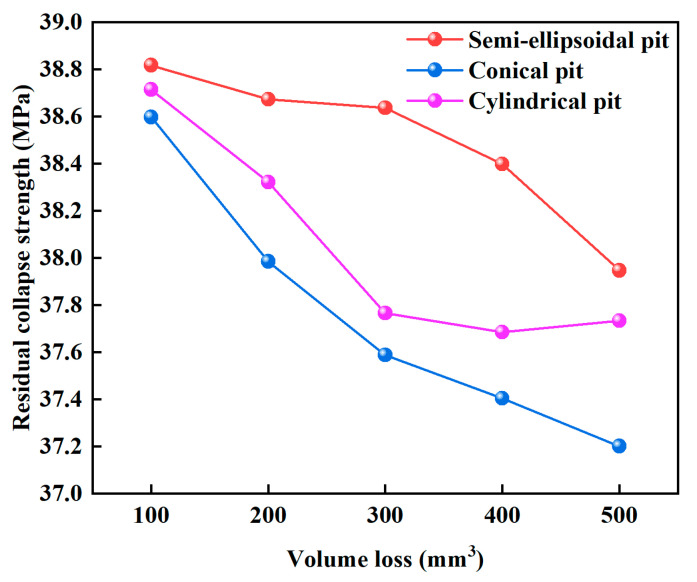
Influence of volume loss on the collapse strength of casing.

As shown in Figure 10, collapse strength decreased with increasing pit volume. Cylindrical pits caused the most severe strength degradation, while semi-ellipsoidal pits had the least impact. When pit volume increased from 100 mm^3^ to 500 mm^3^, cylindrical pits led to a 3.62% reduction in collapse strength. This confirms that collapse strength degradation is not determined by a single factor, but is influenced by a combination of pit depth, pit geometry, and volume loss.

### 3.5. Prediction of Residual Service Life

Based on field data, the casings at different depths are subjected to external pressures of 20 MPa, 11 MPa, and 2 MPa, corresponding to temperatures of 80 °C, 50 °C, and 20 °C, respectively. The widely used mathematical model for engineering prediction of pitting depth is a power-law equation [30]:

(2) $$h=At^{n}$$

where *h* is the corrosion depth (mm), *t* is the exposure time (h), and *A* and *n* are fitting parameters.

**Figure 11:**
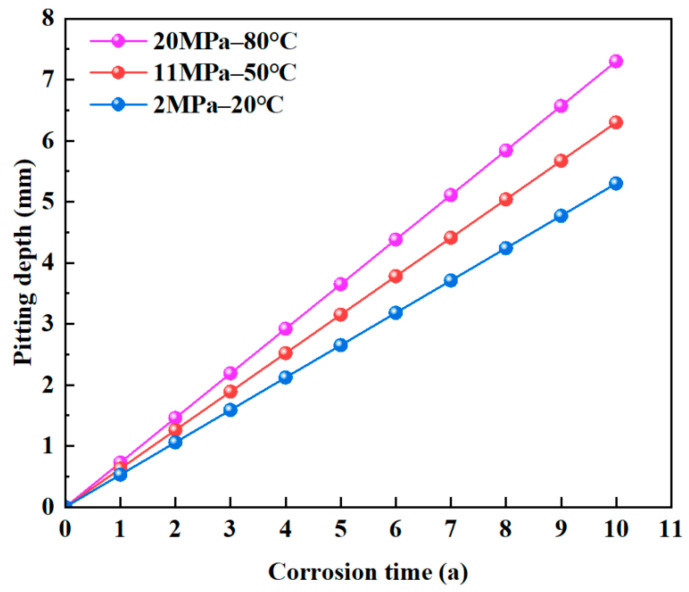
Evolution of corrosion depth with corrosion time.

Based on the results of the hanging coupon corrosion tests and the power-law corrosion model, the relationships between pit depth and corrosion time under three operating conditions were fitted, as shown in Figure 11. As corrosion time increases, the pit depth grows progressively, leading to an increase in the stress concentration factor. Under the same corrosion duration, higher pressure and temperature result in deeper pits.

**Figure 12:**
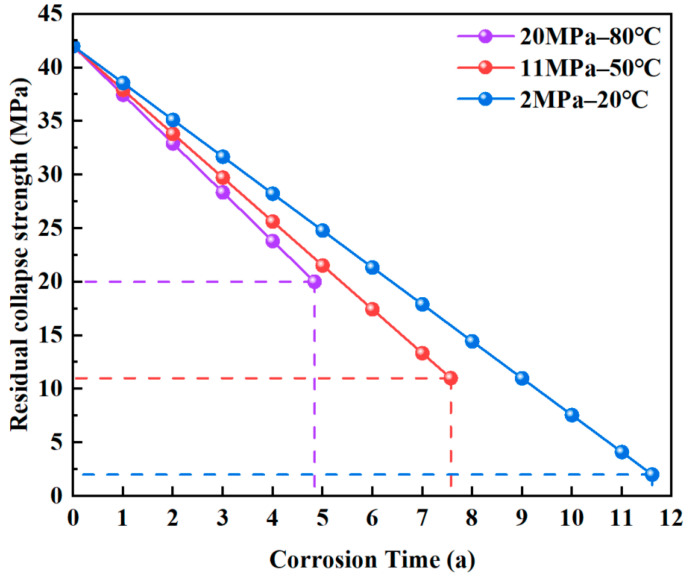
Evolution of casing collapse strength with corrosion time.

Since cylindrical pits have the most significant weakening effect on the collapse strength of the casing, all corrosion pits were assumed to be cylindrical in the subsequent analysis. Using the finite element results, the relationship between residual collapse strength and corrosion time was established, as shown in Figure 12. When the residual collapse strength degrades to the level of the corresponding external pressure, the casing can no longer withstand the load, and this point in time is defined as the end of its service life. The predicted safe service lives under the three conditions are 4.84 years, 7.57 years, and 11.61 years, respectively.

**Figure 13:**
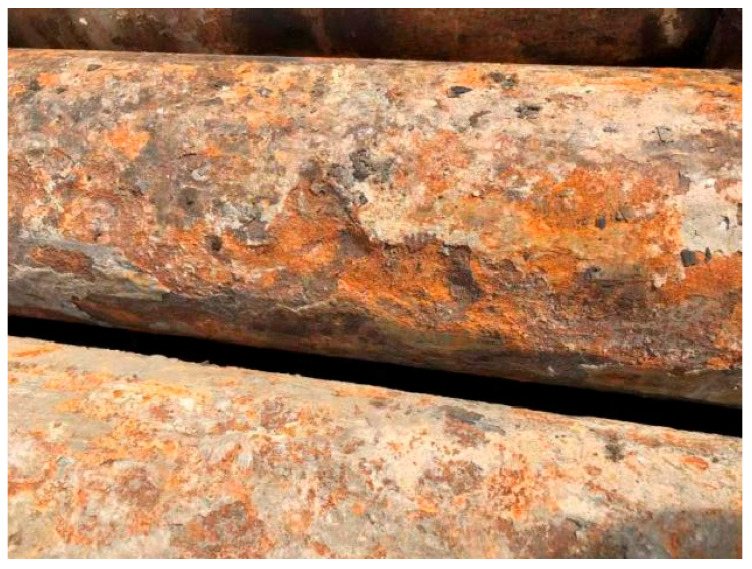
Corrosion condition of casing after four years of service.

Based on the actual corrosion conditions of in-service casings in the Yanchang Oilfield, as shown in Figure 13, some casings exhibited severe uniform and localized corrosion after approximately four years of service, leading to a deterioration in mechanical properties and an inability to meet production requirements. In contrast, this study predicts a safe service life of 4.84 years under the 20 MPa–80 °C condition, which appears slightly conservative. The primary reason for this discrepancy lies in the fact that the actual service environment is more complex, where corrosion behavior is influenced by multiple factors such as temperature fluctuations, in situ stress variations [4], and microbial activity [1]. These factors accelerate the corrosion process, particularly aggravating the severity of localized corrosion. Therefore, although the model prediction is somewhat conservative, it still provides valuable guidance for practical engineering applications.

## 4. Conclusions

This study utilized corrosion testing and finite element modeling to elucidate the degradation mechanism of casing collapse strength in formation water environments and predicted safe service life under field conditions. The main conclusions are as follows:

(1) Elevated temperature and pressure significantly accelerate casing corrosion and reduce collapse strength. Under the 20 MPa–80 °C condition, the corrosion rate increased by 87.5% compared to the 2 MPa–20 °C condition, with greater pit depth and density, leading to a 1.3% reduction in collapse strength.
(2) The collapse strength of casing is influenced by multiple factors, including corrosion pit shape, pit depth, and the volume loss induced by corrosion. As pit depth increases, the collapse strength decreases accordingly. Under the same pit depth, the collapse strength declines with increasing volume loss. Cylindrical pits result in the greatest volume loss and thus the most severe reduction in collapse strength, whereas conical pits cause the least volume loss and have the weakest detrimental effect on collapse resistance.
(3) By combining corrosion test data with finite element simulations, the safe service life of casing was predicted for different well conditions. The predicted lifespans were 4.84 years, 7.57 years, and 11.61 years under 80 °C–20 MPa, 50 °C–11 MPa, and 20 °C–2 MPa conditions, respectively.

## Figures and Tables

**Table 1:** Chemical composition of J55 casing steel (wt.%).

C	Si	Mn	P	S	Cu	Ni	Cr	Mo	Nb	V	Ti
0.18	0.17	0.90	0.014	0.005	0.013	0.013	0.028	0.006	0.013	<0.005	0.0019

**Table 2:** Mechanical properties of J55 casing steel.

Steel	Yield Strength (MPa)	Tensile Strength (MPa)	Elongation	Young’s Modulus (GPa)	Poisson’s Ratio	HRC
J55	388	630	31%	200	0.3	14.5

**Table 3:** Corrosion test conditions.

Pressure (MPa)	Temperature (°C)	Duration (Days)	Specimen Type
2	20	7	Hanging coupon specimen
11	50	7	
20	80	7	
2	20	30	Full-scale casing
11	50	30	
20	80	30	

**Table 4:** Results of the hanging coupon corrosion test.

Pressure (MPa)	Temperature (°C)	Average Corrosion Rate (mm/a)	Maximum Pit Depth (μm)	Pitting Density (m^−2^)
2	20	0.008	10	7 × 10^6^
11	50	0.015	13	7 × 10^6^
20	80	0.017	13	8 × 10^6^

**Table 5:** Comparison between experimental and finite element results of casing collapse strength.

Pressure (MPa)	Temperature (°C)	Experimental Result (MPa)	Finite Element Result (MPa)	Error (%)
2	20	43.93	42.51	−3.2
11	50	43.77	42.12	−3.8
20	80	43.37	41.96	−3.3

## Data Availability

The data presented in this study are available on request from the corresponding author due to privacy.
